# First-line nivolumab plus ipilimumab or chemotherapy versus chemotherapy alone in advanced esophageal squamous cell carcinoma: a Japanese subgroup analysis of open-label, phase 3 trial (CheckMate 648/ONO-4538-50)

**DOI:** 10.1007/s10388-022-00970-1

**Published:** 2022-11-19

**Authors:** Ken Kato, Yuichiro Doki, Takashi Ogata, Satoru Motoyama, Hisato Kawakami, Masaki Ueno, Takashi Kojima, Yasuhiro Shirakawa, Morihito Okada, Ryu Ishihara, Yutaro Kubota, Carlos Amaya-Chanaga, Tian Chen, Yasuhiro Matsumura, Yuko Kitagawa

**Affiliations:** 1 Department of Head and Neck, Esophageal Medical Oncology, National Cancer Center Hospital, Chuo City, Tokyo 104-0045 Japan; 2 Department of Surgery, Osaka University Graduate School of Medicine, Osaka, Japan; 3 Department of Gastrointestinal Surgery, Kanagawa Cancer Center, Yokohama, Japan; 4 Department of Thoracic Surgery, Akita University Graduate School of Medicine, Akita, Japan; 5 Department of Medical Oncology, Kindai University Faculty of Medicine, Osaka-sayama, Japan; 6 Department of Gastroenterological Surgery, Toranomon Hospital, Tokyo, Japan; 7 Gastrointestinal Oncology Division, National Cancer Center Hospital East, Kashiwa, Japan; 8 Department of Gastroenterological Surgery, Graduate School of Medicine, Dentistry and Pharmaceutical Sciences, Okayama University, Okayama, Japan; 9 Department of Surgery, Hiroshima City Hiroshima Citizens Hospital, Hiroshima, Japan; 10 Department of Surgical Oncology, Hiroshima University Hospital, Hiroshima, Japan; 11 Department of Gastrointestinal Oncology, Osaka International Cancer Institute, Osaka, Japan; 12 Department of Medicine, Division of Medical Oncology, Showa University Hospital, Tokyo, Japan; 13 Bristol Myers Squibb, Princeton, NJ USA; 14 Department of Oncology, Ono Pharmaceutical Company Ltd., Osaka, Japan; 15 Department of Surgery, Keio University School of Medicine, Tokyo, Japan

**Keywords:** Esophageal squamous cell carcinoma, Nivolumab, Ipilimumab, Japanese population, First-line treatment

## Abstract

**Background:**

Programmed cell death 1 (PD-1)-based treatments are approved for several cancers. CheckMate 648, a global, phase 3 trial, showed that first-line nivolumab (anti-PD-1 antibody) plus ipilimumab (NIVO + IPI) or nivolumab plus chemotherapy (NIVO + Chemo) significantly increased survival in advanced esophageal squamous cell carcinoma (ESCC) without new safety signals versus chemotherapy alone (Chemo).

**Methods:**

We evaluated the Japanese subpopulation of CheckMate 648 (*n* = 394/970), randomized to receive first-line NIVO + IPI, NIVO + Chemo, or Chemo. Efficacy endpoints included overall survival (OS) and progression-free survival assessed by blinded independent central review in Japanese patients with tumor-cell programmed death-ligand 1 (PD-L1) expression ≥ 1% and in all randomized Japanese patients.

**Results:**

In the Japanese population, 131, 126, and 137 patients were treated with NIVO + IPI, NIVO + Chemo, and Chemo, and 66, 62, and 65 patients had tumor-cell PD-L1 ≥ 1%, respectively. In patients with tumor-cell PD-L1 ≥ 1%, median OS was numerically longer with NIVO + IPI (20.2 months; hazard ratio [95% CI], 0.46 [0.30–0.71]) and NIVO + Chemo (17.3 months; 0.53 [0.35–0.82]) versus Chemo (9.0 months). In all randomized patients, median OS was numerically longer with NIVO + IPI (17.6 months; 0.68 [0.51–0.92]) and NIVO + Chemo (15.5 months; 0.73 [0.54–0.99]) versus Chemo (11.0 months). Grade 3–4 treatment-related adverse events were reported in 37%, 49%, and 36% of all patients in the NIVO + IPI, NIVO + Chemo, and Chemo arms, respectively.

**Conclusion:**

Survival benefits with acceptable tolerability observed for NIVO + IPI and NIVO + Chemo treatments strongly support their use as a new standard first-line treatment in Japanese patients with advanced ESCC.

**ClinicalTrials.gov ID:**

NCT03143153.

**Supplementary Information:**

The online version contains supplementary material available at 10.1007/s10388-022-00970-1.

## Introduction

Esophageal squamous cell carcinoma (ESCC) is the dominant (~ 85%) histological subtype of esophageal cancer globally [1]. In 2018, the age-standardized incidence rate of ESCC per 100,000 person-years was 5 times higher in Japan than in America [1]. ESCC has a poor prognosis, with a 5-year relative survival rate of ~ 40% in 2016, and 1-year relative survival rate of 45% in patients treated with first-line 5-fluorouracil + cisplatin therapy in Japan [2, 3], highlighting the need for more effective treatment options. Until recently, the first-line treatment for advanced ESCC was limited to fluoropyrimidine + platinum-based chemotherapy in Japan [4]. In 2021–2022, the survival benefit of five different first-line treatments with anti-programmed cell death 1 [PD-1] antibodies-plus-chemotherapy over chemotherapy alone was verified by five phase 3 trials, namely KEYNOTE-590 (pembrolizumab) [5], CheckMate 648/ONO-4538-50 (nivolumab) [6], ESCORT-1st (camrelizumab) [7], JUPITER-06 (toripalimab) [8], and ORIENT-15 (sintilimab) [9]. Pembrolizumab + chemotherapy received approval in various countries and has become the standard first-line treatment for ESCC [10]. Based on KEYNOTE-590 results, which included Japanese patients, pembrolizumab + chemotherapy was recommended as the first-line treatment regimen in the Japanese esophageal cancer guidelines [5, 11, 12]. In the global, phase 3, CheckMate 648 study, nivolumab administered along with ipilimumab (anti-cytotoxic T lymphocyte-associated protein 4 antibody [anti-CTLA4 antibody]) or chemotherapy resulted in significantly longer overall survival (OS) versus chemotherapy alone in advanced ESCC [6]. No new safety signals were identified for nivolumab combination therapies [6].

A few phase 3 studies provide evidence for advanced ESCC treatments in the Japanese subgroup. Japanese subpopulation data from phase 3 trials for advanced esophageal cancer are limited to KEYNOTE-181 and ATTRACTION-3 trials that assessed second-line pembrolizumab or nivolumab monotherapy, respectively [13, 14]. Survival outcomes of ESCC were different for Japanese population compared with global clinical trial populations, as seen in ATTRACTION-3, where median OS for nivolumab was numerically longer in the Japanese subpopulation (13.4 months) versus the global intent-to-treat population (10.9 months) [14, 15]. Subsequent anticancer treatment rates were also different between the Japanese and global intent-to-treat population in ATTRACTION-3, suggesting that treatment practices including approval/reimbursement systems may differ between Japan and other countries [3, 14–16]. Therefore, analyzing the efficacy and safety data specific to the Japanese subpopulation from the global phase 3 trials for first-line treatment in patients with advanced ESCC is important. Thus, we evaluated the efficacy and safety of nivolumab combination therapies in the Japanese subpopulation of CheckMate 648, and compared these findings with the global population.

## Methods

### Study design and patients

CheckMate 648 is an open-label, phase 3 trial to assess nivolumab-plus-ipilimumab (NIVO + IPI) and nivolumab-plus-chemotherapy (NIVO + Chemo) combinations compared with chemotherapy alone (Chemo) as a first-line treatment in patients with advanced ESCC. Detailed methods are published elsewhere [6]. Briefly, patients aged ≥ 18 years who had advanced (unresectable, recurrent, or metastatic), histologically confirmed ESCC or adenosquamous cell carcinoma (predominant squamous differentiation) and measurable disease per Response Evaluation Criteria in Solid Tumors (RECIST) version 1.1 were enrolled, irrespective of their PD-L1 expression status. Patients had no prior systemic therapy for advanced disease and were not amenable to cancer-directed curative therapies. Patients were stratified according to tumor-cell PD-L1 expression (PD-L1: ≥ 1% vs. < 1%, or indeterminate), region (East Asia [Japan, Korea, Taiwan] vs. rest of Asia vs. rest of the world), Eastern Cooperative Oncology Group performance status (ECOG PS) (0 vs. 1), and number of organs with metastases (≤ 1 vs. ≥ 2). Eligible patients were randomized (1:1:1) to receive nivolumab (3 mg/kg every 2 weeks) plus ipilimumab (1 mg/kg every 6 weeks), nivolumab (240 mg every 2 weeks) plus chemotherapy (4-week cycle of fluorouracil 800 mg/m^2^ on days 1–5 and cisplatin 80 mg/m^2^ on day 1), or chemotherapy alone (same dose as described above). Treatment continued until disease progression, unacceptable toxicity, consent withdrawal, or end of trial. Nivolumab or NIVO + IPI was administered for up to 2 years in the absence of disease progression or unacceptable toxicity. The current analyses focus on the Japanese racial subpopulation enrolled at Japanese sites, with data cutoff identical to that for the global trial population (January 18, 2021).

### Pre-specified outcomes

The primary endpoints were OS and progression-free survival (PFS) assessed by blinded independent central review (BICR) per RECIST version 1.1 in patients with tumor-cell PD-L1 expression ≥ 1%. Secondary endpoints comprised OS and PFS (BICR) in all randomized patients, and objective response rate (ORR) per BICR in patients with tumor-cell PD-L1 expression ≥ 1% and all randomized patients. Key exploratory endpoints included investigator-assessed PFS, subgroup analyses for OS based on demographics and clinical factors for all patients, duration of response (BICR), and safety. Efficacy endpoints were assessed for the global intent-to-treat population. Adverse events (AEs) were assessed in all the patients who had received ≥ 1 dose of the assigned treatment, and were graded according to the National Cancer Institute Common Terminology Criteria for Adverse Events, v4.0.

### Statistical analyses

Descriptive analyses were conducted for the Japanese subpopulation. Hazard ratios (HRs) for OS and PFS were calculated using unstratified Cox proportional hazards model with treatment as the sole covariate. Two-sided 95% confidence intervals (CIs) for the HR were provided. Median OS, PFS, and duration of response for each treatment arms were estimated using the Kaplan–Meier method, and the corresponding 95% CIs were constructed based on a log–log transformed CI for the survivor function. OS and PFS rates at fixed time points were derived from the Kaplan–Meier estimate. The 95% CIs for proportions were calculated using the Clopper–Pearson method. Statistical analyses were performed using SAS software v9.2 to v9.4 (SAS Institute, Cary, USA) and R statistical software package.

## Results

### Characteristics of the Japanese subpopulation

Overall, 394 of 970 patients from CheckMate 648 trial were enrolled in Japan (NIVO + IPI, *n* = 131; NIVO + Chemo, *n* = 126; Chemo, *n* = 137). Baseline patient characteristics were generally balanced between the treatment arms (Table 1). Of the 394 patients, 386 (98%) received ≥ 1 dose of the study drug (NIVO + IPI, *n* = 130; NIVO + Chemo, *n* = 121; Chemo, *n* = 135). Among the treated patients, the primary reason for treatment discontinuation was disease progression (NIVO + IPI: 55% [71/130]; NIVO + Chemo: 65% [79/121]; Chemo: 69% [93/135]; Online Resource 1). Subsequent therapies were administered to 73% (96/131), 67% (85/126), and 82% (112/137) of the patients in the NIVO + IPI, NIVO + Chemo, and Chemo arms, respectively, with 9.2% (12/131), 9.5% (12/126), and 26.3% (36/137) of the patients receiving subsequent therapy comprising immune checkpoint inhibitors (anti-PD-1 or anti-CTLA4 antibody), respectively (Online Resource 2).

**Table 1:** Baseline demographics and clinical characteristics of the Japanese subpopulation

	NIVO + IPI (*n* = 131)	NIVO + Chemo (*n* = 126)	Chemo (*n* = 137)
Median age, years (range)	66 (34–81)	68 (44–86)	67 (36–78)
Sex, male	111 (84.7)	99 (78.6)	121 (88.3)
ECOG performance status			
0	93 (71.0)	89 (70.6)	95 (69.3)
1	38 (29.0)	37 (29.4)	42 (30.7)
Histology at initial diagnosis			
Squamous cell carcinoma	130 (99.2)	126 (100.0)	137 (100.0)
Adenosquamous carcinoma	1 (0.8)	0	0
Tumor-cell PD-L1 expression^a^			
≥ 1%	66 (51.6)	62 (49.2)	65 (47.4)
< 1%	62 (48.4)	64 (50.8)	71 (51.8)
Indeterminate	3 (2.3)	0	1 (0.7)
Disease status at study entry			
De novo metastatic	78 (59.5)	62 (49.2)	72 (52.6)
Recurrent—locoregional	5 (3.8)	6 (4.8)	7 (5.1)
Recurrent—distant	35 (26.7)	42 (33.3)	37 (27.0)
Un-resectable advanced	13 (9.9)	16 (12.7)	21 (15.3)
Number of organs with metastases			
≤ 1	62 (47.3)	54 (42.9)	60 (43.8)
≥ 2	69 (52.7)	72 (57.1)	77 (56.2)
Smoking status			
Current or former smoker	120 (91.6)	109 (86.5)	120 (87.6)
Never	11 (8.4)	17 (13.5)	17 (12.4)

Data are presented as number (%) of patients unless otherwise mentioned

*Chemo* chemotherapy, *ECOG* Eastern Cooperative Oncology Group, *IPI* ipilimumab, *NIVO* nivolumab, *PD-L1* programmed death-ligand 1

^a^Per test results from interactive web response system data

### Efficacy in the Japanese subpopulation

#### Analyses of the NIVO + IPI versus Chemo arm

**Fig. 1:**
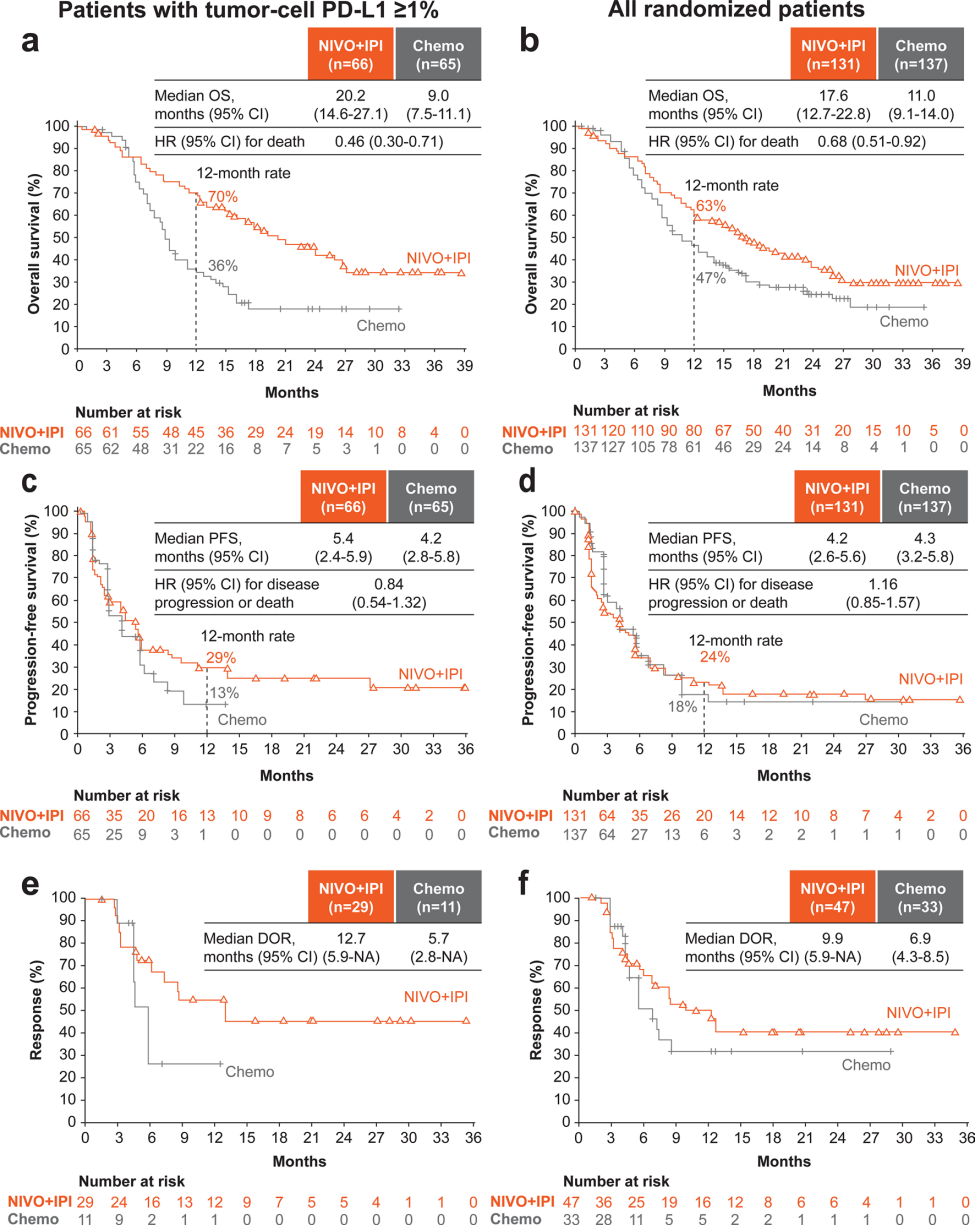
Overall survival, progression-free survival per BICR, and duration of response per BICR with NIVO + IPI versus Chemo in patients with tumor-cell PD-L1 ≥ 1% (left panel: **a**, **c**, **e**) and all randomized patients (right panel: **b**, **d**, **f**) in the Japanese subpopulation. *BICR* blinded independent central review, *Chemo* chemotherapy, *DOR* duration of response, *HR* hazard ratio, *IPI* ipilimumab, *NIVO* nivolumab, *OS* overall survival, *PFS* progression-free survival, *PD-L1* programmed death-ligand 1. Note: Kaplan–Meier estimates are shown, and HRs were calculated using unstratified Cox proportional hazard regression model. DOR was calculated for patients whose best overall response was complete or partial

OS was numerically longer for the NIVO + IPI versus the Chemo arm in patients with tumor-cell PD-L1 ≥ 1% and in all randomized patients. In patients with tumor-cell PD-L1 ≥ 1%, NIVO + IPI had an OS benefit: the median OS increased by 11.2 months versus Chemo (20.2 vs. 9.0 months [refer to the figures for 95% CIs; hereinafter the same]), reducing the risk of death by 54% (HR 0.46 [0.30–0.71]) (Fig. 1a). The corresponding 12-month OS rate doubled for the NIVO + IPI (70%) versus the Chemo (36%) arm. In all randomized patients, NIVO + IPI had numerically longer median OS versus Chemo (17.6 months vs. 11.0 months; HR 0.68 [0.51–0.92]); the 12-month OS rate was 63% versus 47%, respectively (Fig. 1b). In patients with tumor-cell PD-L1 < 1%, median OS was comparable between NIVO + IPI and Chemo (14.5 vs. 14.2 months; HR 1.01 [0.66–1.54]) (Online Resource 3). Median PFS (BICR) in patients with tumor-cell PD-L1 ≥ 1% was numerically longer in the NIVO + IPI versus the Chemo arm (5.4 vs. 4.2 months); however, the risk of progression or death was comparable between the arms (HR 0.84 [0.54–1.32]) (Fig. 1c). In all randomized patients, median PFS (BICR) was comparable between NIVO + IPI versus Chemo arm (4.2 vs. 4.3 months; HR 1.16 [0.85–1.57]) (Fig. 1d). In patients with tumor-cell PD-L1 < 1%, median PFS was 3.2 months in NIVO + IPI versus 5.6 months in Chemo (HR 1.51 [95% CI 0.99–2.31]) (Online Resource 3). The HR trends for PFS per investigator assessment and BICR were similar; per investigator assessment, NIVO + IPI showed PFS benefit versus Chemo in patients with tumor-cell PD-L1 ≥ 1% (5.4 vs. 3.0 months; HR 0.57 [0.38–0.87]) and was comparable with Chemo in all randomized patients (4.1 vs. 4.2 months; HR 0.84 [0.64–1.11]) (Online Resource 4). ORR per BICR was higher, with the complete response rate ~ 4 times higher for NIVO + IPI versus Chemo in patients with tumor-cell PD-L1 ≥ 1% and in all randomized patients (Table 2). Objective (complete or partial) responses were also more durable for NIVO + IPI versus Chemo in patients with tumor-cell PD-L1 ≥ 1% and in all randomized patients (Fig. 1e and f).

**Table 2:** Antitumor activity per blinded independent central review based on RECIST version 1.1 in the Japanese subpopulation

	Patients with tumor-cell PD-L1 expression ≥ 1%			All randomized patients		
	NIVO + IPI (*n* = 66)	NIVO + Chemo (*n* = 62)	Chemo (*n* = 65)	NIVO + IPI (*n* = 131)	NIVO + Chemo (*n* = 126)	Chemo (*n* = 137)
ORR, *n* (%) [95% CI]^a^	29 (43.9) [31.7–56.7]	40 (64.5) [51.3–76.3]	11 (16.9) [8.8–28.3]	47 (35.9) [27.7–44.7]	71 (56.3) [47.2–65.2]	33 (24.1) [17.2–32.1]
Median time to response, months (range)^a^	1.45 (1.2–8.4)	1.51 (0.6–3.1)	1.54 (1.4–4.3)	1.48 (1.2–8.4)	1.51 (0.6–6.8)	1.54 (1.4–4.3)
BOR, *n* (%)						
Complete response	14 (21.2)	16 (25.8)	3 (4.6)	20 (15.3)	25 (19.8)	6 (4.4)
Partial response	15 (22.7)	24 (38.7)	8 (12.3)	27 (20.6)	46 (36.5)	27 (19.7)
Stable disease	17 (25.8)	12 (19.4)	35 (53.8)	36 (27.5)	34 (27.0)	72 (52.6)
Progressive disease	18 (27.3)	8 (12.9)	12 (18.5)	43 (32.8)	15 (11.9)	20 (14.6)

*BOR* best overall response, *Chemo* chemotherapy, *IPI* ipilimumab, *NIVO* nivolumab, *ORR* objective response rate, *PD-L1* programmed death-ligand 1, *RECIST* response evaluation criteria in solid tumors

^a^Comprised patients whose BOR was complete or partial

#### Analyses of the NIVO + Chemo versus Chemo arm

**Fig. 2:**
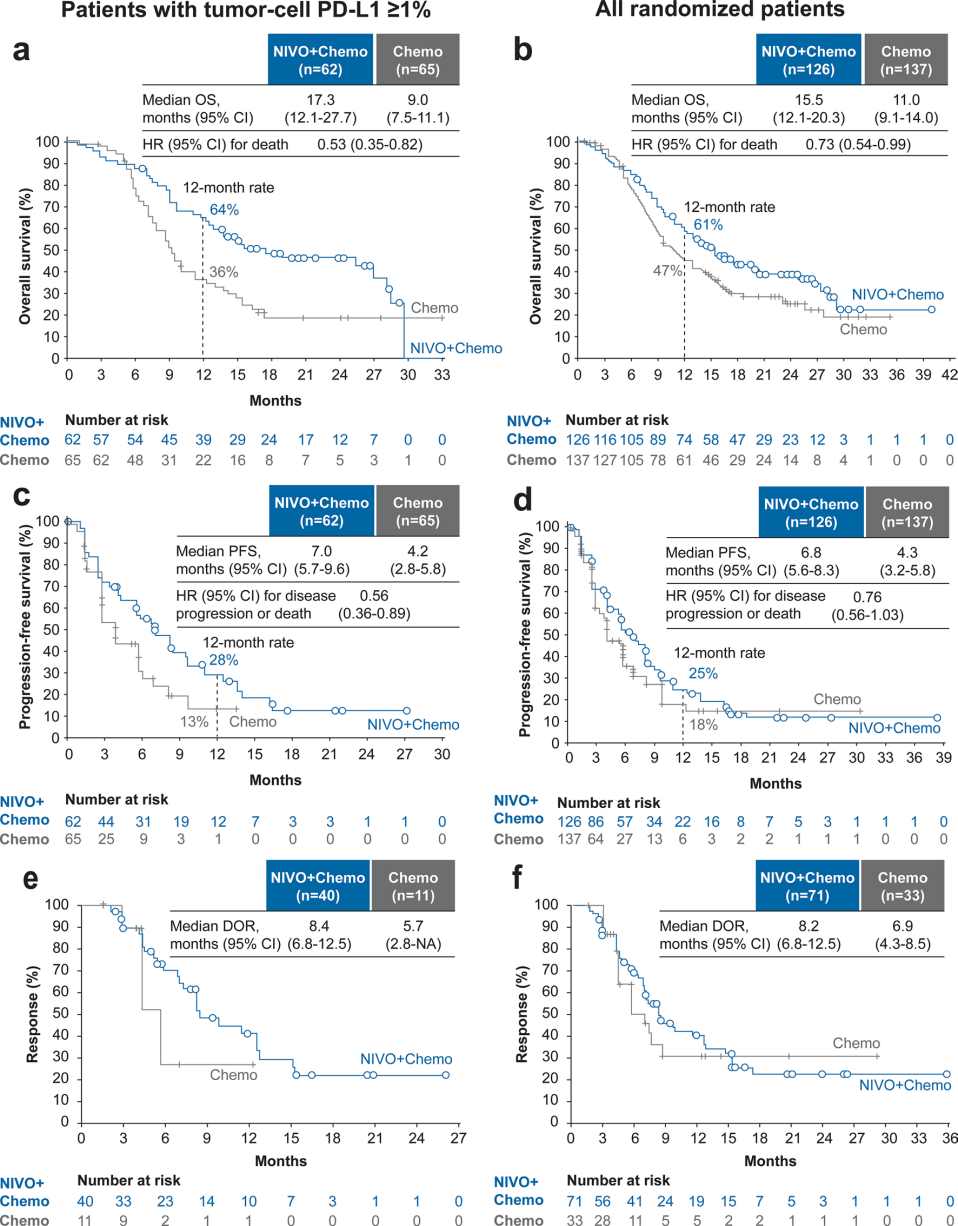
Overall survival, progression-free survival per BICR, and duration of response per BICR with NIVO + Chemo versus Chemo in patients with tumor-cell PD-L1 ≥ 1% (left panel: **a**, **c**, **e**) and all randomized patients (right panel: **b**, **d**, **f**) in the Japanese subpopulation. *BICR* blinded independent central review, *Chemo* chemotherapy, *DOR* duration of response, *HR* hazard ratio, *NIVO* nivolumab, *OS* overall survival, *PFS* progression-free survival, *PD-L1* programmed death-ligand 1. Note: Kaplan–Meier estimates are shown, and HRs were calculated using unstratified Cox proportional hazard regression model. DOR was calculated for patients whose best overall response was complete or partial

In patients with tumor-cell PD-L1 ≥ 1%, NIVO + Chemo numerically increased the median OS versus Chemo (17.3 vs. 9.0 months) and reduced the risk of death by 47% (HR 0.53 [0.35–0.82]); the 12-month OS rate was 64% versus 36%, respectively (Fig. 2a). In all randomized patients, NIVO + Chemo extended the median OS by 4.5 months versus Chemo (15.5 vs. 11.0 months), reducing the risk of death by 27% (HR 0.73 [0.54–0.99]); the 12-month OS rate also increased (61% vs. 47%, respectively) (Fig. 2b). In patients with tumor-cell PD-L1 < 1%, median OS was comparable between NIVO + Chemo and Chemo (14.4 vs. 14.2 months; HR 0.99 [0.65–1.51]) (Online Resource 3). PFS benefit (BICR) was observed in patients with tumor-cell PD-L1 ≥ 1% with NIVO + Chemo versus Chemo (median PFS, 7.0 vs. 4.2 months; HR 0.56 [0.36–0.89]) (Fig. 2c). Though the median PFS was numerically higher in the NIVO + Chemo versus the Chemo arm in all randomized patients (6.8 vs. 4.3 months), the risk of progression or death was not different between the two arms (HR 0.76 [0.56–1.03]) (Fig. 2d). In patients with tumor-cell PD-L1 < 1%, median PFS (BICR) was comparable between NIVO + Chemo and Chemo (5.8 vs. 5.6 months; HR 0.96 [0.63–1.47]) (Online Resource 3). By investigator assessment, NIVO + Chemo showed PFS benefit versus Chemo in patients with tumor-cell PD-L1 ≥ 1% (8.0 vs. 3.0 months; HR, 0.36 [0.23–0.56]) and in all randomized patients (6.8 vs. 4.2 months; HR 0.58 [0.44–0.76]) (Online Resource 4). ORR per BICR was higher in the NIVO + Chemo arm than in the Chemo arm, with the complete response rate increasing > 5 times for patients with tumor-cell PD-L1 ≥ 1% and > 4 times for all randomized patients (Table 2). The duration of response was also longer in the NIVO + Chemo arm than in the Chemo arm for both, tumor-cell PD-L1 ≥ 1% cases and all randomized patients (Fig. 2e and f).

#### Subgroup analyses of OS in all randomized Japanese patients

Subgroup analyses were performed for age, sex, ECOG PS, tumor-cell PD-L1 expression status, disease status at study entry, number of organs with metastases, and smoking history using unstratified Cox proportional hazards modeling with treatment as the only covariate (Online Resource 5). OS numerically favored NIVO + IPI or NIVO + Chemo versus Chemo alone across multiple prespecified subgroups in all randomized patients. In both, NIVO + IPI and NIVO + Chemo arms, the OS prolongation effect was numerically larger with the higher tumor-cell PD-L1 expression subgroup, with no further enrichment of efficacy observed in any of the cutoff values higher than 1% (Online Resource 5).

### Exposure and safety in all-treated Japanese patients

The median treatment duration was longer with NIVO + Chemo (5.6 months), while the values were similar for NIVO + IPI (2.7 months) and Chemo (2.9 months) (Online Resource 6). Among the treated patients, 48 (37%), 59 (49%), and 49 (36%) patients reported grade 3–4 treatment-related AEs (TRAEs) in the NIVO + IPI, NIVO + Chemo, and Chemo arms, respectively; correspondingly, serious TRAEs of any grade occurred in 55 (42%), 25 (21%), and 16 (12%) patients (Table 3). TRAEs led to treatment discontinuation in 32 (25%), 44 (36%), and 32 (24%) patients in the NIVO + IPI, NIVO + Chemo, and Chemo arms, respectively (Table 3). TRAEs led to death in 1 (1%), 2 (2%), and 0 patients in the NIVO + IPI, NIVO + Chemo, and Chemo arms, respectively (Table 3). Most TRAEs with potential immunologic etiology were grade 1–2; grade 3–4 events occurred in ≤ 10% of the patients across organ categories (Online Resource 7).

**Table 3:** Treatment-related adverse events in all treated patients who received ≥ 1 dose of the assigned treatment in the Japanese subpopulation

All treated patients	NIVO + IPI (*n* = 130)^a^		NIVO + Chemo (*n* = 121)^a^		Chemo (*n* = 135)^a^	
	Any grade	Grade 3–4	Any grade	Grade 3–4	Any grade	Grade 3–4
Any TRAE	110 (84.6)	48 (36.9)	120 (99.2)	59 (48.8)	126 (93.3)	49 (36.3)
Serious TRAE	55 (42.3)	36 (27.7)	25 (20.7)	18 (14.9)	16 (11.9)	12 (8.9)
TRAE leading to discontinuation	32 (24.6)	23 (17.7)	44 (36.4)	9 (7.4)	32 (23.7)	4 (3.0)
Treatment-related death^b^	1 (0.8)		2 (1.7)		0 (0)	
TRAEs by preferred term observed in ≥ 10% of patients in any treatment arm						
Rash	23 (17.7)	4 (3.1)	5 (4.1)	1 (0.8)	3 (2.2)	0
Pruritus	22 (16.9)	2 (1.5)	9 (7.4)	0	1 (0.7)	0
Alopecia	0	0	22 (18.2)	0	26 (19.3)	0
Hypothyroidism	22 (16.9)	0	6 (5.0)	0	0	0
Diarrhea	12 (9.2)	0	21 (17.4)	0	24 (17.8)	3 (2.2)
Stomatitis	10 (7.7)	0	64 (52.9)	8 (6.6)	44 (32.6)	0
Nausea	5 (3.8)	0	71 (58.7)	2 (1.7)	71 (52.6)	3 (2.2)
Constipation	1 (0.8)	0	38 (31.4)	2 (1.7)	37 (27.4)	1 (0.7)
Pyrexia	16 (12.3)	0	6 (5.0)	0	1 (0.7)	0
Fatigue	9 (6.9)	0	20 (16.5)	1 (0.8)	15 (11.1)	2 (1.5)
Malaise	9 (6.9)	0	42 (34.7)	1 (0.8)	40 (29.6)	0
Hiccups	2 (1.5)	0	34 (28.1)	0	40 (29.6)	0
Blood creatinine increased	3 (2.3)	0	15 (12.4)	0	16 (11.9)	1 (0.7)
Platelet count decreased	3 (2.3)	0	20 (16.5)	0	16 (11.9)	2 (1.5)
White blood cell count decreased	1 (0.8)	0	27 (22.3)	7 (5.8)	23 (17.0)	6 (4.4)
Creatinine renal	0	0	17 (14.0)	0	7 (5.2)	1 (0.7)
Neutrophil count decreased	0	0	41 (33.9)	17 (14.0)	40 (29.6)	21 (15.6)
Decreased appetite	8 (6.2)	3 (2.3)	72 (59.5)	9 (7.4)	81 (60.0)	6 (4.4)
Hyponatremia	3 (2.3)	2 (1.5)	18 (14.9)	10 (8.3)	11 (8.1)	7 (5.2)
Dysgeusia	4 (3.1)	0	14 (11.6)	0	16 (11.9)	0
Peripheral sensory	1 (0.8)	0	19 (15.7)	0	11 (8.1)	0
Anemia	1 (0.8)	1 (0.8)	29 (24.0)	12 (9.9)	25 (18.5)	8 (5.9)

*TRAE* treatment-related adverse event, *Chemo* chemotherapy, *IPI* ipilimumab, *NIVO* nivolumab

Data are presented as number (%) of patients in each arm

^a^Includes events reported between the first dose and 30 days after the last dose of trial therapy. Treatment-relatedness in the NIVO + Chemo arm was attributed to either nivolumab or any of the chemotherapies or both. Treatment-relatedness in the NIVO + IPI arm was attributed to either nivolumab or ipilimumab or both. TRAEs were reported using National Cancer Institute Common Terminology Criteria for Adverse Events, version 4.0, and Medical Dictionary for Regulatory Activities, version 23.1

^b^Treatment-related deaths were reported regardless of timeframe. Treatment-related death in the NIVO + IPI arm was from pulmonary embolism. Treatment-related deaths in the NIVO + Chemo arm were from pneumatosis intestinalis and pneumonitis

## Discussion

First-line NIVO + IPI and NIVO + Chemo treatments resulted in a substantial survival advantage over Chemo in Japanese patients with advanced ESCC. The efficacy and safety of NIVO + IPI and NIVO + Chemo were consistent between the CheckMate 648 Japanese subpopulation and the global population (including Japan) [6]. For NIVO + IPI and NIVO + Chemo, the median OS and median PFS (BICR) were numerically longer, with higher ORR for the Japanese subpopulation versus the global population. Specifically, in the chemotherapy-free combination (NIVO + IPI), the Japanese subpopulation had numerically longer median OS than the global population (17.6 [12.7–22.8] vs. 12.7 [11.3–15.5] months) [6]. Baseline patient characteristics of the Japanese subpopulation and the global population were generally similar. However, more patients in the Japanese subpopulation had an ECOG PS of 0 in the NIVO + IPI (71% vs. 46%), NIVO + Chemo (71% vs. 47%), and Chemo (69% vs. 48%) arms versus the global population [6]. It is plausible that a better functional status may have contributed to the survival benefit in the Japanese subpopulation compared with the global population. This hypothesis is supported by findings of NIVO + IPI phase 3 trials for non-small cell lung cancer (NSCLC) suggesting that better performance status was correlated with greater NIVO + IPI efficacy [17, 18].

Patients with better clinical condition are generally more likely to receive subsequent treatment. This may explain the higher rates of subsequent anticancer therapies in the current study across all arms in the Japanese subpopulation (63%–75%) versus the global population (51–63%), especially the rate of subsequent systemic anticancer therapies received [6]. Differences in ECOG PS scores and subsequent treatment rates may have affected the OS benefit observed. These findings are concordant with a phase 3 nivolumab monotherapy versus chemotherapy trial (ATTRACTION-3) in advanced ESCC [14, 15]. In ATTRACTION-3, compared with the global population, the Japanese subpopulation had higher rates of ECOG PS 0 (nivolumab, 61% vs. 48%; Chemo, 64% vs. 51%), subsequent systemic anticancer therapies (nivolumab, 59% vs. 53%; Chemo, 47% vs. 47%), and longer median OS (nivolumab, 13.4 vs. 10.9 months; Chemo, 9.4 vs. 8.4 months) [14, 15]. Subsequent chemotherapy after anti-PD-1/PD-L1 therapy improved efficacy outcomes in other types of cancers [19–21], which may have contributed to the higher efficacy of the nivolumab combination treatments in the Japanese subpopulation in this study.

Also, the Japanese subpopulation had a relatively higher percentage of smokers than the global population (89% vs. 80%) [6]. The subgroup analyses of CheckMate 648 global population suggested a longer OS in smokers versus nonsmokers in the NIVO + IPI arm [6]. In contrast, nonsmokers tended to have longer OS in the NIVO + Chemo arm, possibly because the concomitant chemotherapy may override any advantage of nivolumab in smokers. Reportedly, immune checkpoint inhibitors are more effective in patients with a smoking history in NSCLC and several other cancers [22, 23]. These results suggest that the difference in the proportion of smokers may have enhanced the efficacy of NIVO + IPI in the Japanese population.

The HRs for death for NIVO + IPI or NIVO + Chemo versus Chemo were < 1, suggesting overall survival advantage irrespective of increasing tumor-cell PD-L1 cutoff values (≥ 1%, ≥ 5%, ≥ 10%; HRs 0.46, 0.45, 0.50 for NIVO + IPI and 0.54, 0.51, 0.54 for NIVO + Chemo, respectively). In contrast, HRs for death in NIVO + IPI or NIVO + Chemo arms versus Chemo were close to 1 for tumor-cell PD-L1 < 1% (HR 1.01 for NIVO + IPI and 0.99 for NIVO + Chemo, respectively). The relatively smaller efficacy of NIVO + IPI or NIVO + Chemo at tumor-cell PD-L1 < 1% suggests that tumor-cell PD-L1 expression might be one of the predictors of response; however, the magnitude of efficacy did not increase in patients with higher tumor-cell PD-L1 expression levels (≥ 1%, ≥ 5%, ≥ 10%). Therefore, there seems to be a limitation to performing response prediction with only PD-L1.

In the four anti-PD-1 phase 3 studies, namely, KEYNOTE-590 (pembrolizumab), ESCORT-1st, (camrelizumab), JUPITER-06 (toripalimab), and ORIENT-15 (sintilimab), the anti-PD-1 antibody + chemotherapy combination significantly prolonged median OS compared with placebo + chemotherapy [5, 7–9]. Similar findings were noted for the NIVO + Chemo arm in both the Japanese (current study) and global population of the CheckMate 648 trial [6].

In the NIVO + IPI arm, the incidence of serious TRAEs was higher than that in the NIVO + Chemo and Chemo arms, whereas the incidence of any TRAEs was lower. Similar trends were observed in the global population [6]. However, in the NIVO + IPI arm, TRAEs with potential immunologic etiology of endocrine, pulmonary, and skin were slightly more common in the Japanese than in the global population [6]. This concurs with reports on renal cell carcinoma or NSCLC treated with NIVO + IPI, where rates of these AE categories were similarly higher in the Japanese or Asian population than in the global population [24–26].

The NIVO + IPI and NIVO + Chemo arms were not compared statistically as this study was not designed to compare these 2 arms. It was also not possible to speculate advantage of one or the other nivolumab combination treatment for specific study subgroups. In clinical practice, the choice of cancer treatment regimen is governed by several considerations including disease status, patients’ requirements and preferences, and capacity to tolerate specific immunotherapy or chemotherapy. Future studies and exploratory post hoc analyses are warranted to determine prognostic predictors for each of the nivolumab combination regimen.

## Conclusion

NIVO + IPI and NIVO + Chemo in the Japanese subpopulation showed efficacy benefits compared with Chemo and had an acceptable safety profile, similar to reports from the global population. This Japanese sub-analysis showed that both NIVO + IPI and NIVO + Chemo treatments can become a new standard first-line treatment option for Japanese patients with advanced ESCC.

## Supplementary Information

Below is the link to the electronic supplementary material.

Supplementary file1 (PDF 27 KB)

Supplementary file2 (PDF 38 KB)

Supplementary file3 (PDF 102 KB)

Supplementary file4 (PDF 69 KB)

Supplementary file5 (TIF 12456 KB)

Supplementary file6 (PDF 21 KB)

Supplementary file7 (PDF 23 KB)
