# Supplementary material for: First-line nivolumab plus ipilimumab or chemotherapy versus chemotherapy alone in advanced esophageal squamous cell carcinoma: a Japanese subgroup analysis of open-label, phase 3 trial (CheckMate 648/ONO-4538-50)
Source: Esophagus. 2022 Nov 19;20(2):291–301. doi: 10.1007/s10388-022-00970-1 (PMC10024660; doi:10.1007/s10388-022-00970-1)
Supplement: Supplementary file 1 — Supplementary file1 (PDF 27 KB) [file 10388_2022_970_MOESM1_ESM.pdf]

Online Resource 1

Table S1 Patients discontinuing treatment and reasons for discontinuation among the treated Japanese patients

	**NIVO + IPI** (n=130)	**NIVO + Chemo** (n=121)	**Chemo** (n=135)
Discontinued treatment	123 (94.6)	112 (92.6)	135 (100)
Reasons for treatment discontinuation			
Disease progression	71 (54.6)	79 (65.3)	93 (68.9)
AE related to treatment	32 (24.6)	12 (9.9)	22 (16.3)
AE not related to treatment	6 (4.6)	8 (6.6)	5 (3.7)
Patient request	3 (2.3)	4 (3.3)	5 (3.7)
Other[a]	11 (8.5)	9 (7.4)	10 (7.4)

AE, adverse event; Chemo, chemotherapy; IPI, ipilimumab; NIVO, nivolumab.

Data are presented as number (%) of patients in each arm.

[a]Includes death, pregnancy, consent withdrawal, maximum clinical benefit, treatment completion as per protocol, and other.

Journal: *Esophagus (Original article)*

Manuscript title

First-line nivolumab plus ipilimumab or chemotherapy versus chemotherapy alone in advanced esophageal squamous cell carcinoma: a Japanese subgroup analysis of open-label, phase 3 trial (CheckMate 648/ONO-4538-50)

Authors

Ken Kato[1], Yuichiro Doki[2], Takashi Ogata[3], Satoru Motoyama[4], Hisato Kawakami[5], Masaki Ueno[6], Takashi Kojima[7], Yasuhiro Shirakawa[8,9], Morihito Okada[10], Ryu Ishihara[11], Yutaro Kubota[12], Carlos Amaya-Chanaga[13], Tian Chen[13], Yasuhiro Matsumura[14], Yuko Kitagawa[15]

[1]Department of Head and Neck, Esophageal Medical Oncology, National Cancer Center Hospital, Tokyo, Japan

[2]Department of Surgery, Osaka University Graduate School of Medicine, Osaka, Japan

[3]Department of Gastrointestinal Surgery, Kanagawa Cancer Center, Yokohama, Japan

[4]Department of Thoracic Surgery, Akita University Graduate School of Medicine, Akita, Japan

[5]Department of Medical Oncology, Kindai University Faculty of Medicine, Osaka-sayama, Japan

[6]Department of Gastroenterological Surgery, Toranomon Hospital, Tokyo, Japan

[7]Gastrointestinal Oncology Division, National Cancer Center Hospital East, Kashiwa, Japan

[8]Department of Gastroenterological Surgery, Graduate School of Medicine, Dentistry and Pharmaceutical Sciences, Okayama University, Okayama, Japan

[9]Department of Surgery, Hiroshima City Hiroshima Citizens Hospital, Hiroshima, Japan

[10]Department of Surgical Oncology, Hiroshima University Hospital, Hiroshima, Japan

[11]Department of Gastrointestinal Oncology, Osaka International Cancer Institute, Osaka, Japan

[12]Department of Medicine, Division of Medical Oncology, Showa University Hospital, Tokyo, Japan

[13]Bristol Myers Squibb, Princeton, NJ, USA

[14]Department of Oncology, Ono Pharmaceutical Company Ltd., Osaka, Japan

[15]Department of Surgery, Keio University School of Medicine, Tokyo, Japan

Corresponding author: Ken Kato

Department of Head and Neck, Esophageal Medical Oncology, National Cancer Center Hospital, Chuo City, Tokyo 104-0045, Japan

Phone: (+)81-3-3542-2511; Email: kenkato@ncc.go.jp
