# Supplementary material for: First-line nivolumab plus ipilimumab or chemotherapy versus chemotherapy alone in advanced esophageal squamous cell carcinoma: a Japanese subgroup analysis of open-label, phase 3 trial (CheckMate 648/ONO-4538-50)
Source: Esophagus. 2022 Nov 19;20(2):291–301. doi: 10.1007/s10388-022-00970-1 (PMC10024660; doi:10.1007/s10388-022-00970-1)
Supplement: Supplementary file 2 — Supplementary file2 (PDF 38 KB) [file 10388_2022_970_MOESM2_ESM.pdf]

Online Resource 2

Table S2 Subsequent therapies administered in the Japanese subpopulation

	**NIVO + IPI** (n=131)	**NIVO + Chemo** (n=126)	**Chemo** (n=137)
Any subsequent therapy	96 (73.3)	85 (67.5)	112 (81.8)
Radiotherapy	49 (37.4)	43 (34.1)	61 (44.5)
Curative	4 (3.1)	7 (5.6)	6 (4.4)
Palliative	45 (34.4)	37 (29.4)	55 (40.1)
Surgery	1 (0.8)	5 (4.0)	6 (4.4)
Curative	1 (0.8)	1 (0.8)	3 (2.2)
Palliative	0	4 (3.2)	3 (2.2)
Systemic therapy	86 (65.6)	79 (62.7)	103 (75.2)
Anti-PD-1	11 (8.4)	12 (9.5)	36 (26.3)
Nivolumab	11 (8.4)	12 (9.5)	34 (24.8)
BI 754091	0	0	1 (0.7)
Pembrolizumab	0	0	2 (1.5)
Anti-CTLA-4	1 (0.8)	0	0
Ipilimumab	1 (0.8)	0	0
Other	84 (64.1)	78 (61.9)	93 (67.9)
Fluorouracil	78 (59.5)	24 (19.0)	48 (35.0)
Cisplatin	74 (56.5)	18 (14.3)	33 (24.1)
Paclitaxel	33 (25.2)	53 (42.1)	52 (38.0)
Docetaxel	18 (13.7)	29 (23.0)	30 (21.9)
Gimeracil, oteracil potassium, tegafur	10 (7.6)	14 (11.1)	16 (11.7)
Nedaplatin	8 (6.1)	13 (10.3)	11 (8.0)
Oxaliplatin	5 (3.8)	4 (3.2)	9 (6.6)
Bevacizumab	1 (0.8)	0	0

Gemcitabine hydrochloride	1 (0.8)	0	0
Lorlatinib	1 (0.8)	0	0
Ramucirumab	1 (0.8)	0	0
BI 754111	0	0	1 (0.7)
Calcium levofolinate	0	1 (0.8)	0
Carboplatin	0	0	1 (0.7)
Cetuximab	0	0	1 (0.7)
Folinic acid	0	0	1 (0.7)
Investigational antineoplastic drugs	0	0	3 (2.2)
Irinotecan	0	0	1 (0.7)
OBP-301	0	0	1 (0.7)

Chemo, chemotherapy; CTLA-4, cytotoxic T-lymphocyte-associated protein-4; IPI, ipilimumab; NIVO, nivolumab; PD-1, programmed death-1.

Data are presented as number (%) of the patients in each arm.

Journal: *Esophagus (Original article)*

Manuscript title

First-line nivolumab plus ipilimumab or chemotherapy versus chemotherapy alone in advanced esophageal squamous cell carcinoma: a Japanese subgroup analysis of open-label, phase 3 trial (CheckMate 648/ONO-4538-50)

Authors

Ken Kato[1], Yuichiro Doki[2], Takashi Ogata[3], Satoru Motoyama[4], Hisato Kawakami[5], Masaki Ueno[6], Takashi Kojima[7], Yasuhiro Shirakawa[8,9], Morihito Okada[10], Ryu Ishihara[11], Yutaro Kubota[12], Carlos Amaya-Chanaga[13], Tian Chen[13], Yasuhiro Matsumura[14], Yuko Kitagawa[15]

[1]Department of Head and Neck, Esophageal Medical Oncology, National Cancer Center Hospital, Tokyo, Japan

[2]Department of Surgery, Osaka University Graduate School of Medicine, Osaka, Japan

[3]Department of Gastrointestinal Surgery, Kanagawa Cancer Center, Yokohama, Japan

[4]Department of Thoracic Surgery, Akita University Graduate School of Medicine, Akita, Japan

[5]Department of Medical Oncology, Kindai University Faculty of Medicine, Osaka-sayama, Japan

[6]Department of Gastroenterological Surgery, Toranomon Hospital, Tokyo, Japan

[7]Gastrointestinal Oncology Division, National Cancer Center Hospital East, Kashiwa, Japan

[8]Department of Gastroenterological Surgery, Graduate School of Medicine, Dentistry and Pharmaceutical Sciences, Okayama University, Okayama, Japan

[9]Department of Surgery, Hiroshima City Hiroshima Citizens Hospital, Hiroshima, Japan

[10]Department of Surgical Oncology, Hiroshima University Hospital, Hiroshima, Japan

[11]Department of Gastrointestinal Oncology, Osaka International Cancer Institute, Osaka, Japan

[12]Department of Medicine, Division of Medical Oncology, Showa University Hospital, Tokyo, Japan

[13]Bristol Myers Squibb, Princeton, NJ, USA

[14]Department of Oncology, Ono Pharmaceutical Company Ltd., Osaka, Japan

[15]Department of Surgery, Keio University School of Medicine, Tokyo, Japan

Corresponding author: Ken Kato

Department of Head and Neck, Esophageal Medical Oncology, National Cancer Center Hospital, Chuo City, Tokyo 104-0045, Japan

Phone: (+)81-3-3542-2511; Email: kenkato@ncc.go.jp
