# Supplementary material for: First-line nivolumab plus ipilimumab or chemotherapy versus chemotherapy alone in advanced esophageal squamous cell carcinoma: a Japanese subgroup analysis of open-label, phase 3 trial (CheckMate 648/ONO-4538-50)
Source: Esophagus. 2022 Nov 19;20(2):291–301. doi: 10.1007/s10388-022-00970-1 (PMC10024660; doi:10.1007/s10388-022-00970-1)
Supplement: Supplementary file 3 — Supplementary file3 (PDF 102 KB) [file 10388_2022_970_MOESM3_ESM.pdf]

**Online Resource 3**

**Table S3 Overall survival and progression-free survival per BICR with tumor-cell PD-L1 <1%**

| | **NIVO + IPI** (n=62) | **NIVO + Chemo** (n=64) | **Chemo** (n=72) |
|---|---|---|---|
| Overall survival | | | |
| Median overall survival, months (95% CI) | 14.5 (10.1–19.7) | 14.4 (10.3–19.7) | 14.2 (10.8–19.5) |
| Hazard ratio (95% CI)[a] | 1.01 (0.66–1.54) | 0.99 (0.65–1.51) | - |
| Progression-free survival | | | |
| Median progression-free survival, months (95% CI) | 3.2 (1.5–5.6) | 5.8 (4.2–8.2) | 5.6 (3.8–8.4) |
| Hazard ratio (95% CI)[a] | 1.51 (0.99–2.31) | 0.96 (0.63–1.47) | - |

BICR, blinded independent central review; Chemo, chemotherapy; IPI, ipilimumab; NIVO, nivolumab.

[a]Computed for each study arm versus Chemo arm.

**Journal:** *Esophagus (Original article)*

**Manuscript title**

First-line nivolumab plus ipilimumab or chemotherapy versus chemotherapy alone in advanced esophageal squamous cell carcinoma: a Japanese subgroup analysis of open-label, phase 3 trial (CheckMate 648/ONO-4538-50)

**Authors**

Ken Kato[1], Yuichiro Doki[2], Takashi Ogata[3], Satoru Motoyama[4], Hisato Kawakami[5], Masaki Ueno[6], Takashi Kojima[7], Yasuhiro Shirakawa[8,9], Morihito Okada[10], Ryu Ishihara[11], Yutaro Kubota[12], Carlos Amaya-Chanaga[13], Tian Chen[13], Yasuhiro Matsumura[14], Yuko Kitagawa[15]

[1]Department of Head and Neck, Esophageal Medical Oncology, National Cancer Center Hospital, Tokyo, Japan

[2]Department of Surgery, Osaka University Graduate School of Medicine, Osaka, Japan

[3]Department of Gastrointestinal Surgery, Kanagawa Cancer Center, Yokohama, Japan

[4]Department of Thoracic Surgery, Akita University Graduate School of Medicine, Akita, Japan

[5]Department of Medical Oncology, Kindai University Faculty of Medicine, Osaka-sayama, Japan

[6]Department of Gastroenterological Surgery, Toranomon Hospital, Tokyo, Japan

[7]Gastrointestinal Oncology Division, National Cancer Center Hospital East, Kashiwa, Japan

[8]Department of Gastroenterological Surgery, Graduate School of Medicine, Dentistry and Pharmaceutical Sciences, Okayama University, Okayama, Japan

[9]Department of Surgery, Hiroshima City Hiroshima Citizens Hospital, Hiroshima, Japan

[10]Department of Surgical Oncology, Hiroshima University Hospital, Hiroshima, Japan

[11]Department of Gastrointestinal Oncology, Osaka International Cancer Institute, Osaka, Japan

[12]Department of Medicine, Division of Medical Oncology, Showa University Hospital, Tokyo, Japan

[13]Bristol Myers Squibb, Princeton, NJ, USA

[14]Department of Oncology, Ono Pharmaceutical Company Ltd., Osaka, Japan

[15]Department of Surgery, Keio University School of Medicine, Tokyo, Japan

**Corresponding author**: Ken Kato

Department of Head and Neck, Esophageal Medical Oncology, National Cancer

Center Hospital, Chuo City, Tokyo 104-0045, Japan

Phone: (+)81-3-3542-2511; Email: kenkato@ncc.go.jp
