# Supplementary material for: First-line nivolumab plus ipilimumab or chemotherapy versus chemotherapy alone in advanced esophageal squamous cell carcinoma: a Japanese subgroup analysis of open-label, phase 3 trial (CheckMate 648/ONO-4538-50)
Source: Esophagus. 2022 Nov 19;20(2):291–301. doi: 10.1007/s10388-022-00970-1 (PMC10024660; doi:10.1007/s10388-022-00970-1)
Supplement: Supplementary file 4 — Supplementary file4 (PDF 69 KB) [file 10388_2022_970_MOESM4_ESM.pdf]

Online Resource 4

Table S4 Progression-free survival per investigator assessment in the Japanese subpopulation

	**NIVO + IPI**		**NIVO + Chemo**		**Chemo**	
	Tumor-cell PD-L1 ≥1% (n=66)	All (n=131)	Tumor-cell PD-L1 ≥1% (n=62)	All (n=126)	Tumor-cell PD-L1 ≥1% (n=65)	All (n=137)
Median PFS, months (95% CI)	5.42 (2.89-5.85)	4.11 (2.79-5.55)	8.02 (5.65-9.07)	6.77 (5.36-8.15)	3.02 (2.76-4.17)	4.17 (3.02-5.32)
Hazard ratio (95% CI)[a]	0.57 (0.38-0.87)	0.84 (0.64-1.11)	0.36 (0.23-0.56)	0.58 (0.44-0.76)	-	-

Chemo, chemotherapy; IPI, ipilimumab; NIVO, nivolumab; PD-L1, programmed death-ligand 1; PFS, progression-free survival.

[a]Computed for each study arm versus Chemo arm.

Journal: *Esophagus (Original article)*

Manuscript title

First-line nivolumab plus ipilimumab or chemotherapy versus chemotherapy alone in advanced esophageal squamous cell carcinoma: a Japanese subgroup analysis of open-label, phase 3 trial (CheckMate 648/ONO-4538-50)

Authors

Ken Kato[1], Yuichiro Doki[2], Takashi Ogata[3], Satoru Motoyama[4], Hisato Kawakami[5], Masaki Ueno[6], Takashi Kojima[7], Yasuhiro Shirakawa[8,9], Morihito Okada[10], Ryu Ishihara[11], Yutaro Kubota[12], Carlos Amaya-Chanaga[13], Tian Chen[13], Yasuhiro Matsumura[14], Yuko Kitagawa[15]

[1]Department of Head and Neck, Esophageal Medical Oncology, National Cancer Center Hospital, Tokyo, Japan

[2]Department of Surgery, Osaka University Graduate School of Medicine, Osaka, Japan

[3]Department of Gastrointestinal Surgery, Kanagawa Cancer Center, Yokohama, Japan

[4]Department of Thoracic Surgery, Akita University Graduate School of Medicine, Akita, Japan

[5]Department of Medical Oncology, Kindai University Faculty of Medicine, Osaka-sayama, Japan

[6]Department of Gastroenterological Surgery, Toranomon Hospital, Tokyo, Japan

[7]Gastrointestinal Oncology Division, National Cancer Center Hospital East, Kashiwa, Japan

[8]Department of Gastroenterological Surgery, Graduate School of Medicine, Dentistry and Pharmaceutical Sciences, Okayama University, Okayama, Japan

[9]Department of Surgery, Hiroshima City Hiroshima Citizens Hospital, Hiroshima, Japan

[10]Department of Surgical Oncology, Hiroshima University Hospital, Hiroshima, Japan

[11]Department of Gastrointestinal Oncology, Osaka International Cancer Institute, Osaka, Japan

[12]Department of Medicine, Division of Medical Oncology, Showa University Hospital, Tokyo, Japan

[13]Bristol Myers Squibb, Princeton, NJ, USA

[14]Department of Oncology, Ono Pharmaceutical Company Ltd., Osaka, Japan

[15]Department of Surgery, Keio University School of Medicine, Tokyo, Japan

Corresponding author: Ken Kato

Department of Head and Neck, Esophageal Medical Oncology, National Cancer Center Hospital, Chuo City, Tokyo 104-0045, Japan

Phone: (+)81-3-3542-2511; Email: kenkato@ncc.go.jp
