# Supplementary material for: First-line nivolumab plus ipilimumab or chemotherapy versus chemotherapy alone in advanced esophageal squamous cell carcinoma: a Japanese subgroup analysis of open-label, phase 3 trial (CheckMate 648/ONO-4538-50)
Source: Esophagus. 2022 Nov 19;20(2):291–301. doi: 10.1007/s10388-022-00970-1 (PMC10024660; doi:10.1007/s10388-022-00970-1)
Supplement: Supplementary file 6 — Supplementary file6 (PDF 21 KB) [file 10388_2022_970_MOESM6_ESM.pdf]

Online Resource 6

Table S5 Duration of treatment and dose modifications in all treated Japanese patients

Treatment arms and specific drugs	Median duration of treatment, months (range)	Dose reduction, number (%)	Dose delays, number (%)
**NIVO + IPI** (n=130)	2.7 (0.0-24.0)		
Nivolumab	2.7 (0.0-24.0)	-	57 (43.8)
Ipilimumab	2.1 (0.0-24.0)	-	48 (36.9)
**NIVO + Chemo** (n=121)	5.6 (0.1-23.8)		
Nivolumab	5.6 (0.0-23.8)	-	90 (74.4)
Cisplatin	3.8 (0.0-13.6)	62 (51.2)	72 (59.5)
Fluorouracil	4.6 (0.1-23.0)	28 (23.1)	79 (65.3)
**Chemo** (n=135)	2.9 (0.0-14.9)		
Cisplatin	2.3 (0.0-14.8)	44 (32.6)	53 (39.3)
Fluorouracil	2.9 (0.1-14.9)	13 (9.8)	56 (42.1)

Chemo, chemotherapy; IPI, ipilimumab; NIVO, nivolumab.

Journal: *Esophagus (Original article)*

Manuscript title

First-line nivolumab plus ipilimumab or chemotherapy versus chemotherapy alone in advanced esophageal squamous cell carcinoma: a Japanese subgroup analysis of open-label, phase 3 trial (CheckMate 648/ONO-4538-50)

Authors

Ken Kato[1], Yuichiro Doki[2], Takashi Ogata[3], Satoru Motoyama[4], Hisato Kawakami[5], Masaki Ueno[6], Takashi Kojima[7], Yasuhiro Shirakawa[8,9], Morihito Okada[10], Ryu Ishihara[11], Yutaro Kubota[12], Carlos Amaya-Chanaga[13], Tian Chen[13], Yasuhiro Matsumura[14], Yuko Kitagawa[15]

[1]Department of Head and Neck, Esophageal Medical Oncology, National Cancer Center Hospital, Tokyo, Japan

[2]Department of Surgery, Osaka University Graduate School of Medicine, Osaka, Japan

[3]Department of Gastrointestinal Surgery, Kanagawa Cancer Center, Yokohama, Japan

[4]Department of Thoracic Surgery, Akita University Graduate School of Medicine, Akita, Japan

[5]Department of Medical Oncology, Kindai University Faculty of Medicine, Osaka-sayama, Japan

[6]Department of Gastroenterological Surgery, Toranomon Hospital, Tokyo, Japan

[7]Gastrointestinal Oncology Division, National Cancer Center Hospital East, Kashiwa, Japan

[8]Department of Gastroenterological Surgery, Graduate School of Medicine, Dentistry and Pharmaceutical Sciences, Okayama University, Okayama, Japan

[9]Department of Surgery, Hiroshima City Hiroshima Citizens Hospital, Hiroshima, Japan

[10]Department of Surgical Oncology, Hiroshima University Hospital, Hiroshima, Japan

[11]Department of Gastrointestinal Oncology, Osaka International Cancer Institute, Osaka, Japan

[12]Department of Medicine, Division of Medical Oncology, Showa University Hospital, Tokyo, Japan

[13]Bristol Myers Squibb, Princeton, NJ, USA

[14]Department of Oncology, Ono Pharmaceutical Company Ltd., Osaka, Japan

[15]Department of Surgery, Keio University School of Medicine, Tokyo, Japan

Corresponding author: Ken Kato

Department of Head and Neck, Esophageal Medical Oncology, National Cancer

Center Hospital, Chuo City, Tokyo 104-0045, Japan

Phone: (+)81-3-3542-2511; Email: kenkato@ncc.go.jp
