# Supplementary material for: First-line nivolumab plus ipilimumab or chemotherapy versus chemotherapy alone in advanced esophageal squamous cell carcinoma: a Japanese subgroup analysis of open-label, phase 3 trial (CheckMate 648/ONO-4538-50)
Source: Esophagus. 2022 Nov 19;20(2):291–301. doi: 10.1007/s10388-022-00970-1 (PMC10024660; doi:10.1007/s10388-022-00970-1)
Supplement: Supplementary file 7 — Supplementary file7 (PDF 23 KB) [file 10388_2022_970_MOESM7_ESM.pdf]

Online Resource 7

Table S6 Treatment-related AEs with potential immunologic etiology

AE category	**NIVO + IPI** (n=130)		**NIVO + Chemo** (n=121)		**Chemo** (n=135)	
Select TRAEs[a]	Any grade	Grade 3-4	Any grade	Grade 3-4	Any grade	Grade 3-4
Endocrine	46 (35.4)	13 (10.0)	12 (9.9)	3 (2.5)	0	0
Gastrointestinal	16 (12.3)	1 (0.8)	23 (19.0)	2 (1.7)	25 (18.5)	4 (3.0)
Hepatic	13 (10.0)	7 (5.4)	11 (9.1)	4 (3.3)	4 (3.0)	1 (0.7)
Pulmonary	16 (12.3)	4 (3.1)	7 (5.8)	0	0	0
Renal	3 (2.3)	0	33 (27.3)	1 (0.8)	24 (17.8)	2 (1.5)
Skin	52 (40.0)	5 (3.8)	19 (15.7)	1 (0.8)	6 (4.4)	0
Infusion-related hypersensitivity	7 (5.4)	0	2 (1.7)	0	1 (0.7)	0

AE, adverse event; Chemo, chemotherapy; IPI, ipilimumab; NIVO, nivolumab; TRAEs, treatment-related adverse events.

Data are presented as number (%) of the patients in each arm.

[a]Includes events reported between the first dose and 30 days after the last dose of the trial therapy.

Journal: *Esophagus (Original article)*

Manuscript title

First-line nivolumab plus ipilimumab or chemotherapy versus chemotherapy alone in advanced esophageal squamous cell carcinoma: a Japanese subgroup analysis of open-label, phase 3 trial (CheckMate 648/ONO-4538-50)

Authors

Ken Kato[1], Yuichiro Doki[2], Takashi Ogata[3], Satoru Motoyama[4], Hisato Kawakami[5], Masaki Ueno[6], Takashi Kojima[7], Yasuhiro Shirakawa[8,9], Morihito Okada[10], Ryu Ishihara[11], Yutaro Kubota[12], Carlos Amaya-Chanaga[13], Tian Chen[13], Yasuhiro Matsumura[14], Yuko Kitagawa[15]

[1]Department of Head and Neck, Esophageal Medical Oncology, National Cancer Center Hospital, Tokyo, Japan

[2]Department of Surgery, Osaka University Graduate School of Medicine, Osaka, Japan

[3]Department of Gastrointestinal Surgery, Kanagawa Cancer Center, Yokohama, Japan

[4]Department of Thoracic Surgery, Akita University Graduate School of Medicine, Akita, Japan

[5]Department of Medical Oncology, Kindai University Faculty of Medicine, Osaka-sayama, Japan

[6]Department of Gastroenterological Surgery, Toranomon Hospital, Tokyo, Japan

[7]Gastrointestinal Oncology Division, National Cancer Center Hospital East, Kashiwa, Japan

[8]Department of Gastroenterological Surgery, Graduate School of Medicine, Dentistry and Pharmaceutical Sciences, Okayama University, Okayama, Japan

[9]Department of Surgery, Hiroshima City Hiroshima Citizens Hospital, Hiroshima, Japan

[10]Department of Surgical Oncology, Hiroshima University Hospital, Hiroshima, Japan

[11]Department of Gastrointestinal Oncology, Osaka International Cancer Institute, Osaka, Japan

[12]Department of Medicine, Division of Medical Oncology, Showa University Hospital, Tokyo, Japan

[13]Bristol Myers Squibb, Princeton, NJ, USA

[14]Department of Oncology, Ono Pharmaceutical Company Ltd., Osaka, Japan

[15]Department of Surgery, Keio University School of Medicine, Tokyo, Japan

Corresponding author: Ken Kato

Department of Head and Neck, Esophageal Medical Oncology, National Cancer Center Hospital, Chuo City, Tokyo 104-0045, Japan

Phone: (+)81-3-3542-2511; Email: kenkato@ncc.go.jp
